# Transcatheter aortic valve replacement embolization: A fleeing, formidable, yet defeatable foe

**DOI:** 10.1016/j.xjse.2024.100030

**Published:** 2024-10-12

**Authors:** Maxwell C. Braasch, Ali M. Alakhtar, Alan Zajarias, Tsuyoshi Kaneko

**Affiliations:** a Division of Cardiothoracic Surgery, Department of Surgery, Washington University School of Medicine, St Louis, Mo; b Department of Surgery, College of Medicine, Qassim University, Qassim, Kingdom of Saudi Arabia; c Division of Cardiovascular Medicine, Department of Medicine, Washington University School of Medicine, St Louis, Mo

**Keywords:** transcatheter aortic valve replacement, valve embolization, aortic stenosis

**Figure:**
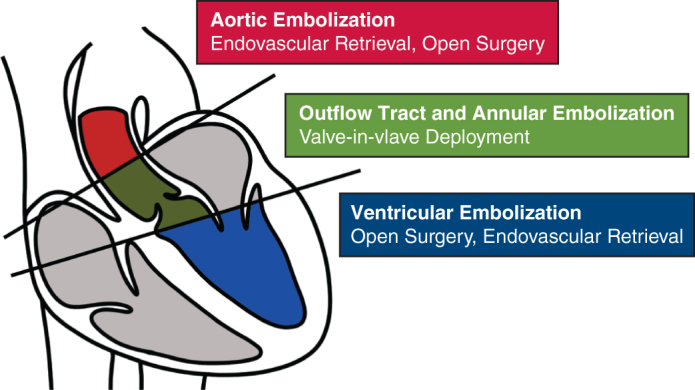
Categorization of TAVR embolization treatment based on location of valve embolization.

**Central Message**

TAVR embolization is a rare phenomenon that clinicians have little to no experience treating. With appropriate knowledge, this potentially emergency complication can be properly assessed and solved.

Transcatheter aortic valve replacement (TAVR) is an innovative treatment for aortic valve pathology. TAVRs are performed by either balloon-expandable (BEV) or self-expandable bioprosthetic valve (SEV) deployment across the aortic valve annulus. Fixation of the valve in place relies upon the radial force applied by the stent framing on the surrounding tissue and anchoring of the valve on rigid structures of the aortic annulus. When the valve loses contact with the annulus and moves from its intended location, embolization occurs.^1^ This is a rare but serious complication of TAVR. With proper prompt management, devastating complications can be prevented.

## Definition and Classification of Valve Embolization

Ectopic valve deployment, valve migration, and valve embolization are subtypes of valve malpositioning.^1^ Deployment of a TAVR valve in a location other than the intended location of the aortic root is ectopic deployment. TAVR valve migration refers to the initially correctly placed TAVR valve moving but still in contact with the aortic annulus. TAVR valve embolization is movement of the prosthesis after final deployment to the degree that it loses contact with the annulus. TAVR valve embolization is categorized as early if it occurs within the first hour of deployment and late if it occurs after this hour.^2^

**Figure 1:**
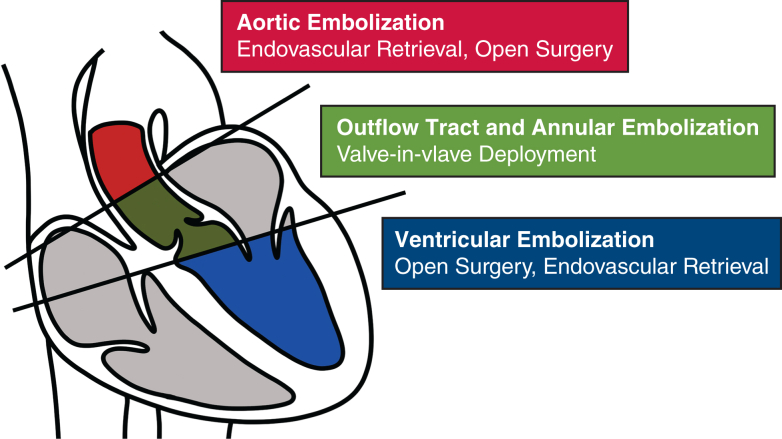
Categorization of transcatheter aortic valve replacement embolization treatment based on location of valve embolization.

We propose that TAVR valve embolization be categorized in 3 ways based on location: aortic, outflow tract/annular, and ventricular embolization (Figure 1). This categorization of embolization is relevant to its treatments.

## Different Valve Types and Valve Embolization

There are 2 types of TAVR valves: SEV and BEV. The first-generation SEV is an independent predictor of embolization.^2^ It is likely that the lack of reproducible deployment and the migration of SEVs during deployment contributed to their embolization. Early trials of a second-generation SEV, the Evolut valve (Medtronic), demonstrated 0 cases of valve embolization in 241 patients.^3^ Evolut has been improved through shortening valve height without shortening the pericardial skirt, improving prevention of paravalvular leak.^4^ Evolut also introduced the recapturing mechanism in which the valve could be resheathed and redeployed after full deployment, accomplished by inverse rotation of the delivery system just before the capsule indicator passing the point of no return. A third-generation SEV, the Evolut Pro (Medtronic), expanded on the Evolut's design by adding an external pericardial wrap. Evolut Pro showed a lower burden of paravalvular leak and only 1 case of valve embolization or migration in 60 patients in the setting of a change of deployment technique as well as valve type.^5^

The Sapien family of valves (Edwards Lifesciences) are BEV and do not have the ability to recapture. Slight differences in design and deployment technique with each valve generation likely lessen the risk of embolization.

## Incidence of Embolization by Era

In the initial US trials of TAVR across high-, intermediate-, and low-risk patients, the incidence of valve embolization ranged from 0.5% to 1%.^6^, ^7^, ^8^ The TranscatheteR HeArt Valve EmboLization and Migration (TRAVEL) Registry published the incidence of embolization in approximately 1% of TAVR procedures from 2010 to 2017.^2^ A 2022 analysis of 226 patients implanted with the fourth-generation SEV, Evolut FX valve (Medtronic) demonstrated 1 (0.4%) valve embolization.^9^ An analysis of 10,312 patients who underwent TAVR with the latest-generation BEV, Sapien 3 Ultra Resilia valve (Edwards Lifesciences), showed embolization occurred in 13 patients (0.1%).^10^ Improvements in SEVs and BEVs contribute to the decrease in the incidence of valve embolization.

## Cause of Valve Embolization

TAVR valve embolization occurs due to patient and technical factors. Patient factors that elevate the risk of valve embolization are lack of calcium on the aortic valve annulus, bicuspid aortic valve, horizontal aorta, and reduced left ventricular (LV) ejection fraction.^2^^,^^11^, ^12^, ^13^ Valve deployment over calcium along the annulus helps anchor the valve in place. Without calcium, a valve may lack sufficient support to anchor in the correct place. Fibrosis of the annulus secondary to valvulitis, which may be appreciated on pre-TAVR imaging with annular thickening and associated myocardial fibrosis, can help prevent embolization. Therefore, aortic regurgitation (AR) without sufficient calcium and fibrosis is a higher risk of valve embolization. A device developed specific to treat AR would likely lessen embolization risk in this setting. Another high-risk group are those with bicuspid valves.^14^ It is more difficult to discern the border between the annulus and the leaflets due to the asymmetric atrioventricular (AV) complex. Many dimensions of the AV complex are larger in bicuspid anatomy, surpassing the upper limit of the largest available valves. The AV complex is nontubular in a large proportion of patients with bicuspid aortic valve anatomy, increasing the possibility of underexpansion or annulus injury during deployment. Supra-annular deployment in patients with tapered anatomy or slight oversizing of the valve in patients with bicuspid valves may help improve valve seating.^14^ Increased aorto-ventricular angulation, known as a horizontal aorta, has been shown to increase the risk of TAVR valve embolization due to difficulties with valve deployment.^13^ Lastly, low LV ejection fraction is associated with TAVR valve embolization, possibly due to rapid valve deployment and limited reimplantation attempts in this population.^12^

Important technical causes of TAVR valve embolization are incorrect valve size,^2^^,^^15^^,^^16^ malpositioning,^2^^,^^13^^,^^15^ pacemaker malcapture,^2^^,^^17^ and postdilation. Errors in sizing or deployment can predispose to valve embolization. With refined techniques to analyze the TAVR size using a dedicated computed tomography scan rather than an echocardiogram, the sizing accuracy has increased, making this error infrequent. Sizing with 3-dimensional transesophageal echocardiography can be used with certainty in patients who cannot receive a contrasted study. Malpositioning can occur from misinterpreting the aortogram during TAVR or an incorrect coplanar view when the aortogram is being performed. The use of the cusp-overlap technique^18^ and consideration of the membranous septum length^19^ may be useful to ensure adequate depth of implantation to avoid embolization but avoid implantation deep enough to cause conduction abnormalities. Pacemaker malcapture, causing missed rapid pacing leading to ventricular ejection, has been indicated as the cause of ∼5% of TAVR embolizations in the TRAVEL registry.^2^ Postdilation of the valve after deployment has been identified as an additional risk factor^12^ that will be discussed in 1 of our proceeding cases.

## Treatment of Valve Embolization

The most common sites for TAVR valve embolization are the ascending aorta at 38%, the LV at 31%, and the descending aorta at 23%.^20^ The method of fixing valve embolization depends on the location of embolization. In general, embolization into the LV requires open surgical retrieval,^2^^,^^19^ although cases of valve migration into LV outflow tract may be salvaged by valve-in-valve anchoring.^15^^,^^21^, ^22^, ^23^ LV embolization has been corrected by valve recapture and redeployment.^24^ Embolization slightly distal to the annular landing zone may also be treated with valve-in-valve anchoring^15^^,^^21^^,^^22^ or, in rare cases, with balloon-retrieval and stenting in the aorta.^16^ Pulling the valve distally into the aorta to accomplish this should be performed carefully because this has been associated with aortic dissection.^25^ Embolization into the ascending aorta should be managed by placing the index valve in the aorta followed by a second TAVR in the appropriate position (Figure 1). Surgical removal of the TAVR valve and surgical AVR is seldom needed for distal embolization; however, should be considered when other options fail. The most critical technical pearl is not removing the wire used for valve deployment. Withdrawal of the wire may lead to the 180° valve rotation, especially for BEV, leading to catastrophic hemodynamic instability. In some instances, mechanical support device placement may be required to assist with temporary hemodynamic instability and acute AR after the second valve is positioned. These treatment recommendations are not absolute, and case-by-case management strategies are required.

## Cases of Valve Embolization

We have experienced 2 interesting cases of TAVR valve embolization in recent years that demonstrate unique fixes to this problem.

### Case #1

**Figure 2:**
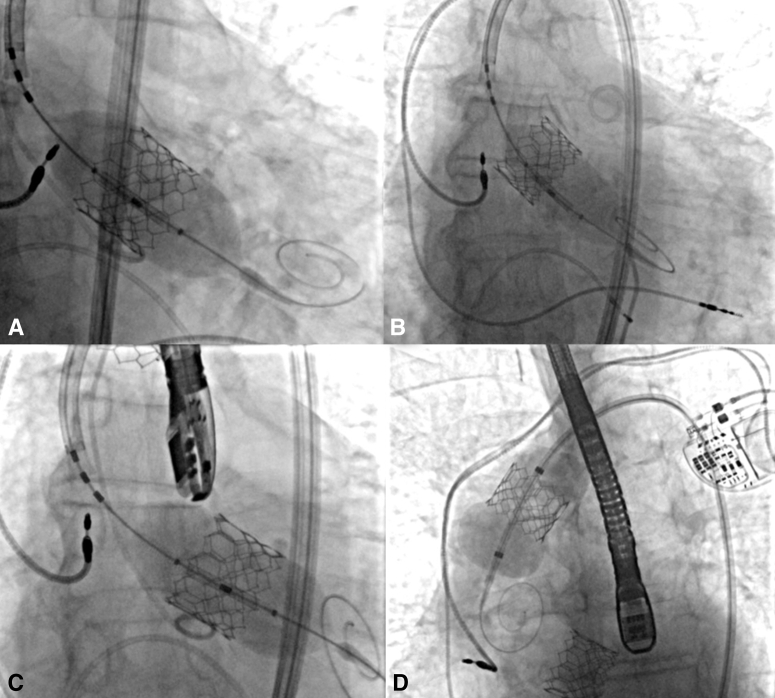
Fluoroscopy images of transcatheter aortic valve replacement (*TAVR*) in case #1. A, Deployment of 26-mm Edwards S3 valve. B, Embolization of TAVR valve into the aortic arch. C, Successful placement of a second 26-mm Edwards S3 valve. D, Unsuccessful attempts to hyperinflate and fixate embolized valve to ascending aorta with 46-mm balloon.

The first case was an 85-year-old man with aortic stenosis (AS) who underwent a transfemoral TAVR with a 26-mm Edwards S3 valve (Figure 2). After deployment, the valve was noted to be slightly high despite adequate ventricular pacing at 180 beats per minute. The valve was postdilated and subsequently embolized distally into the ascending aorta with temporary occlusion of the left coronary ostium. The valve delivery balloon was inflated with an extra 2 cc and used to pull the valve slightly more distal to resolve the ostial obstruction. Next, a second 26-mm Edwards S3 valve was introduced and placed successfully through the embolized valve. Subsequent attempts to hyperexpand and fixate the embolized valve with a 46-mm Coda balloon (Cook Medical) did not produce adequate aortic wall opposition. During attempts to retract the valve with the Coda balloon fully dilated, the balloon and wire pulled free from the valve, allowing the embolized valve to turn 180°, blocking flow through the aorta. Wire access across the embolized valve was then reestablished and an Impella (Abiomed) was then placed on an emergency basis for temporary cardiac support across this embolized valve while emergency surgical retrieval was initiated. This emergency retrieval required a median sternotomy with an aortotomy on cardiopulmonary bypass. His postoperative course was complicated by a seizure, but he fully recovered with no neurological deficit or subsequent seizures and was discharged on hospital day 9. Echocardiography obtained 6 days after TAVR demonstrated no AS or AR.

### Case #2

**Figure 3:**
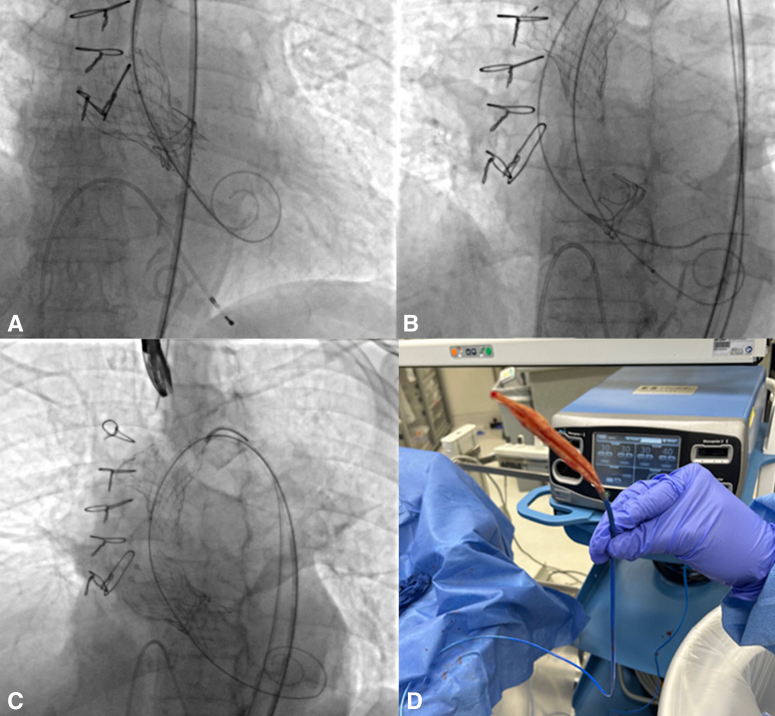
Fluoroscopy and operating room images of transcatheter aortic valve replacement (*TAVR*) case #2. A, Deployment of a 23-mm Evolut FX (Medtronic) valve. B, Embolization of a TAVR valve into the ascending aorta. C, Successful placement of a 23-mm Evolut FX valve. D, Shaft of the 20-mm balloon was cut and removed from the patient after advancement of a 12Fr sheath over the balloon.

The second case was of an 89-year-old woman with severe AS (Figure 3). The 23-mm Evolut FX valve was deployed and postdilatation was performed with a 20-mm True balloon (Becton, Dickinson and Company) for incomplete expansion. However, at the time of balloon removal, there was significant resistance. Multiple strategies, including reinflating/deflating the balloon, additional negative suction did not allow the balloon to be retrieved at the point of stent frame. Then the retraction led to its embolization into the aorta. A second 23-mm Evolut FX valve was then placed without issue. The embolized valve was pulled to the proximal aortic arch, where the interaction with the arch held the valve in place. Ultimately, a 12 Fr Cordis sheath (Cordis) was advanced over the balloon after the shaft of the balloon was cut and the sheath with the balloon was removed. She was discharged to cardiac rehabilitation on hospital day 17. Post-TAVR echocardiography demonstrating no AS or AR.

## Conclusions

Identifying risk factors for TAVR embolization can prepare operators regarding the risk of this complication. Correct valve sizing and precision with deployment cannot be overstated in the importance of minimizing the risk of embolization. Numerous options exist for the treatment of embolized valves after TAVR. It is critical to know the strategies to implement should valve embolization occur.

## Conflict of Interest Statement

Dr Kaneko discloses his relationship as a consultant for Edwards Lifesciences, Medtronic, and 4C Medical, as well as a speaker for Abbott and Baylis. Dr Zajarias discloses a consultant relationship with Edwards Lifesciences, Medtronic, and Anteris. All other authors reported no conflicts of interest.

The *Journal* policy requires editors and reviewers to disclose conflicts of interest and to decline handling or reviewing manuscripts for which they may have a conflict of interest. The editors and reviewers of this article have no conflicts of interest.
